# Electrophysiological monitoring of injury progression in the rat cerebellar cortex

**DOI:** 10.3389/fnsys.2014.00197

**Published:** 2014-10-09

**Authors:** Gokhan Ordek, Archana Proddutur, Vijayalakshmi Santhakumar, Bryan J. Pfister, Mesut Sahin

**Affiliations:** ^1^Department of Biomedical Engineering, New Jersey Institute of TechnologyNewark, NJ, USA; ^2^Department of Neurology and Neurosciences, Rutgers Biomedical and Health SciencesNewark, NJ, USA

**Keywords:** cerebellar evoked potentials, traumatic injury model, Purkinje cells, multi-electrode arrays, micro-ECoG

## Abstract

The changes of excitability in affected neural networks can be used as a marker to study the temporal course of traumatic brain injury (TBI). The cerebellum is an ideal platform to study brain injury mechanisms at the network level using the electrophysiological methods. Within its crystalline morphology, the cerebellar cortex contains highly organized topographical subunits that are defined by two main inputs, the climbing (CFs) and mossy fibers (MFs). Here we demonstrate the use of cerebellar evoked potentials (EPs) mediated through these afferent systems for monitoring the injury progression in a rat model of fluid percussion injury (FPI). A mechanical tap on the dorsal hand was used as a stimulus, and EPs were recorded from the paramedian lobule (PML) of the posterior cerebellum via multi-electrode arrays (MEAs). Post-injury evoked response amplitudes (EPAs) were analyzed on a daily basis for 1 week and compared with pre-injury values. We found a trend of consistently decreasing EPAs in all nine animals, losing as much as 72 ± 4% of baseline amplitudes measured before the injury. Notably, our results highlighted two particular time windows; the first 24 h of injury in the acute period and day-3 to day-7 in the delayed period where the largest drops (~50% and 24%) were observed in the EPAs. In addition, cross-correlations of spontaneous signals between electrode pairs declined (from 0.47 ± 0.1 to 0.35 ± 0.04, *p* < 0.001) along with the EPAs throughout the week of injury. In support of the electrophysiological findings, immunohistochemical analysis at day-7 post-injury showed detectable Purkinje cell loss at low FPI pressures and more with the largest pressures used. Our results suggest that sensory evoked potentials (SEPs) recorded from the cerebellar surface can be a useful technique to monitor the course of cerebellar injury and identify the phases of injury progression even at mild levels.

## Introduction

Assessment of the injury progression in the neural circuits affected by brain trauma is crucial not only to understand the underlying pathophysiological mechanisms but also for designing new therapeutic interventions. However, there is currently no clinical method available to assess the severity of brain injuries acutely and monitor its progression over days following the injury. If certain milestones of injury progression can be identified, therapeutic interventions can be made at specific times targeting parts of the circuit under attack by the secondary mechanism of injury.

Traumatic brain injury (TBI) results from a direct or indirect force exerted on the head that quickly leads to a sequel of changes in the brain such as mechanical tissue deformation, hemorrhage, and an elevated level of intracranial pressure. Cerebellar related deficits were reported in individuals within weeks to years following head injury (Iwadate et al., 1989; Louis et al., 1996). Metabolic changes, which could be an indicator heralding a pathological sequel of head trauma, have also been reported in the cerebellar injuries (Kushner et al., 1987; Niimura et al., 1999; Hattori et al., 2003). Extensive cell death can be observed in different parts of the brain as early as 10 min and progresses over a month following injury (Conti et al., 1998; Sato et al., 2001; Ai et al., 2007). Ai et al. (2007) showed progressive Purkinje cells (PC) loss after direct injury to the rat cerebellum, which may also be correlated with electrophysiological changes within the cerebellar circuitry (Ai and Baker, 2002, 2004). While the initial injury is predominantly dependent on the severity of the impact, subsequent reactions, which may last days to months, involve a complex sequence of events (Thompson et al., 2005; Marklund et al., 2006; Bramlett and Dietrich, 2007). The latter is an emerging field of research, especially regarding under-diagnosed cases such as concussions since they present a broad window of cascaded injury events.

It has been suggested that progression of a brain injury involves molecular and cellular cascaded mechanisms, which may occur in minutes to months after trauma (Thompson et al., 2005; Marklund et al., 2006; Bramlett and Dietrich, 2007). Glutamate, the primary excitatory neurotransmitter in the CNS, is highly utilized in the cerebellum between different cell types; mossy fibers (MFs)—granule cells (GCs), Parallel fibers—PCs and Climbing fibers (CFs)—PCs (Ito, 2001; Nishiyama and Linden, 2007). One of the proposed mechanisms of brain injury involves excessive release of glutamate that leads to excitotoxicity (Gross, 2006). Elevation in the glutamate transmission is likely to disrupt the synaptic communication in the cerebellar cortex and compromise the cerebellar function. Ai et al. documented presynaptic hyperexcitation at the parallel fibers and the amplitude increase in the MF potentials on the days after the injury in the rat cerebellum (Ai and Baker, 2002, 2004). In fluid percussion injury (FPI) rats 7 days after injury, the same investigators also reported an amplitude decrease in the complex spike activity, which typically occurs by direct activation of the CFs on the PCs. (Ai et al., 2007).

The lack of complete understanding of brain injury mechanisms, particularly the mild cases, motivates the search for novel methods of detection and their integration into clinical practice. Brainstem auditory (BAEP), visual (VEP) and somatosensory (SSEP) evoked potentials (EPs) recorded from various areas of the brain with EEG electrodes have been utilized to detect brain injuries in the acute phase (Lindsay et al., 1981; Narayan et al., 1981; Claassen and Hansen, 2001; Nuwer et al., 2005; Burghaus et al., 2013). Sensory evoked potentials (SEPs) are highly reproducible across individuals and they can detect subtle changes in the post-injury period. A typical EP analysis includes comparing peak-to-peak amplitudes and onset latencies of characteristic deflections (Lindsay et al., 1981; Claassen and Hansen, 2001), fluctuations in the power spectrum (Culic et al., 2005; Nuwer et al., 2005; Burghaus et al., 2013), and changes in the coherence spectrum (Thatcher et al., 1989).

Evoked potentials obtained from the cerebellar surface as a response to a stimulus in anesthetized animals have been analyzed to understand the neural signal flow in the cerebellar cortex (Eccles et al., 1967; Oscarsson, 1968; Armstrong and Drew, 1980; Atkins and Apps, 1997; Baker et al., 2001; Diwakar et al., 2011). Eccles et al. (1967) used the juxtafastigial stimulation to identify subsequent MF mediated evoked responses (i.e., P1, N1, P2, N2 and N3) with <5 ms onset latencies in anesthetized cats. Armstrong and Drew (1980) observed these potentials with similar onset latencies associated with MF activation to snout stimulation in surface recordings from the rat cerebellum. They also compared the evoked field potentials of the cerebellum by different depth measurements and stated that all MF-mediated signal components were detectable from the surface using micro-electrodes. In a more recent study, MF-potentials were clearly reported with the denoted onset latencies in ketamine-xylazine treated rats, despite the substantial depression in the evoked amplitudes (Bengtsson and Jörntell, 2007).

Also, due to the divergent connection of MFs on the sole output of the cerebellar cortex, the PCs, the activation of MFs can be detected at multiple levels of the neuronal circuitry. D’Angelo et al. (2001) showed ~5 ms (N2 wave) onset latency for the MF—GC activation to intracerebellar electric stimulation, which was consistent with earlier reports. Adversely, the earliest evoked responses were recorded at ~13 ms (T wave) in the granular layer to a tactile stimulation of the whisker pad. Investigators suggested that the N2 and T waves were directly comparable (Roggeri et al., 2008).

Climbing fibers; one of the two afferents to the cerebellum, contribute significantly to sensory processing, and their EPs can also be detected with surface recordings. In an earlier report, the CF related EPs to a forelimb nerve stimulation was detected at 14–22 ms after the stimulus in the cerebellar surface potentials (Larson et al., 1969). Armstrong et al. (1973) reported observable CF-activation with the onset latencies of 16–22 ms and 20–25 ms in the vermis and ipsilateral hemisphere of the rat cerebellum, respectively. Atkins and Apps conducted a detailed characterization of CF mediated EPs and concluded noticeable variations in the onset latencies of CF field potentials within the same lobule of the rat cerebellum. The local field potentials (LFPs) contained CF activations with 10–15 ms onset latencies to forelimb stimulation in the central area of the paramedian lobule (PML), while CF response delays spread over to 16–26 ms in the lateral side of the PML with forelimb stimulation (Atkins and Apps, 1997). All these reports agree that both MF- and CF-related EPs are detectable from surface recordings. Furthermore, it is convenient to be able to identify these two responses by their onset latencies where the MF-related EPs precede the CF-related responses.

In this study, we recorded SEPs using micro electrocorticography (ECoG) electrode arrays implanted on the rat cerebellar cortex. Detection of subtle changes in the EPs, which are indications of alterations in the underlying neural circuitry, can provide valuable information about the injury development at high spatio-temporal resolution.

## Materials and methods

### Multi-electrode arrays (MEAs) implantation

**Figure 1:**
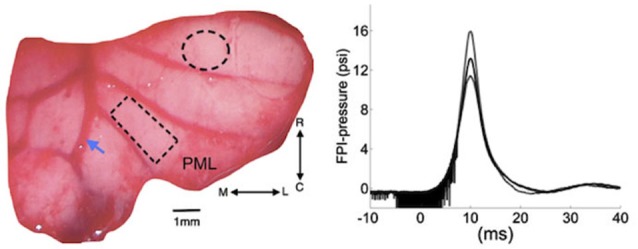
**Left: Position of cranial opening to induce FPI is shown as a dashed circle on the right cerebellar lobe rostral to the multi-electrode array (rectangle) implanted on the paramedian lobule (PML)**. Arrow shows the right paravermal vein. Right: the mean (solid trace) and lowest and highest (dash traces) of the FPI-pressure waves administered in all the rats of this study (*n* = 9). The noise seen before the rising phase is an artifact generated by forward motion of the voice coil. M-L: medio-lateral, R-C: rostro-caudal.

Flexible multi-electrode arrays (MEAs) were chronically implanted in 15 (6-uninjured control for 21 days and 9-injured for 7 days) Sprague-Dawley rats (250–350 g) using sterile surgical techniques. All procedures were approved and performed in accordance to the guidelines of the Institutional Animal Care and Use Committee, Rutgers University, Newark, NJ. The rats were anesthetized with ketamine and xylazine mixture (100 mg/kg and 10 mg/kg respectively, IP), and additional doses were administered as needed during the surgical procedure. The skull over the PML of the cerebellum was removed. A custom-design 31-channel flexible substrate (thickness 12 µm) MEA (NeuroNexus, MI) was placed subdurally on the paramedian cortex (rectangle, Figure 1.), 0.5–1.0 mm lateral to the paravermal vein (Figure 1). Electrode contacts were 50 µm in diameter and at 300 µm from each other in a 4 × 8 configuration. Electrode impedances varied between 300–500 kΩ prior to implantation. Impedances measured between 700–800 kΩ immediately after implantation and stabilized around 500–600 kΩ in the survival period (7 or 21-days). The electrode array was fixed in place to the pia mater using very small amounts of octyl cyanoacrylate tissue adhesive (Nexaband, WPI, Inc., FL) applied on the edges of the array. A reference electrode was placed above the array after covering the dura with a piece of animal’s own connective tissue. The Omnetics micro connector at the end of the ribbon cable was fixed to the skull using dental acrylic and stainless steel screws. Detailed electrode implantation procedure was previously described (Ordek et al., 2013).

### Injury site craniotomy

A craniotomy of 1.8 mm diameter was opened to administer a pressure wave and injure the cerebellum at a distance of 2–2.5 mm from the edge of the implanted electrode (dashed circle, Figure 1). Once the dura was exposed, a plastic male Luer-loc port with a ~1.7 mm inner diameter (cut from a 25G needle) was attached on the skull over the injury site with Nexaband adhesive. The port was filled with normal saline to check for leakage. Then, dental acrylic was applied to firmly attach the injury port to the skull.

### Fluid percussion injury (FPI)

Nine of the 15 animals implanted with the MEAs were injured using the FPI method. The FPI device used in this study was a custom design voice-coil based system that could control the characteristics of the pressure waveform including the rise time, peak pressure and duration (Neuberger et al., 2014). A user specifies the displacement, velocity and acceleration of the voice coil that drives a hydraulic cylinder to generate the pressure wave when it hits the saline column in contact with the animal’s brain. Integrated pressure transducer (Omega Inc.) and LabView program acquired the actual pressure waveform measured near the exit port for later analysis (right, Figure 1). A 2.3 mm-ID female Luer-loc fitting connects to the male Luer-loc cemented to the skull on the experimental animal. Animals were mildly anesthetized with a low dosage of ketamine and xylazine (50 mg/kg and 6 mg/kg respectively, IP). Prior to injury, the dura was visible through the port, which was then filled with 0.2 mL deionized water, free of air bubbles. Next, the animal was placed on a platform attached to the FPI device and a single pulse of pressure wave was released (~15 psi, Figure 1). Only the animals that demonstrated overt signs of injury, i.e., a brief apnea (5–10 s), startling, and deferred-drowsiness (~up to 3 h), were considered as successfully injured (*n* = 9 rats). No functional deficits, difficulty in walking or reaching, were observed in any of the FPI animals during the 7-day survival period.

### Electrophysiology

Injured animals were removed from the FPI device and taken to the recording setup immediately (within 5 min) after the injury pulse to observe very early changes in the cerebellar signals. Recording sessions were kept relatively long on the day of injury (up to 1 h post-FPI) to observe the acute effects of injury. An additional dose of ketamine (30 mg/kg, IP) was administered for these sessions. Anesthesia regimen is very critical to obtain reproducible evoked potentials from the cerebellum and the cerebrum (Bengtsson and Jörntell, 2007; Ordek et al., 2013). Spontaneous and SEPs were collected each day for the next 7 days under anesthesia. The anesthesia dosage (ketamine/xylazine; 30 mg/kg and 2 mg/kg) and time of recording from anesthesia onset (~5 min after) were standardized in order to minimize variations in anesthesia depth between recording sessions.

The recordings (both from injured and control animals) were performed in a large Faraday cage through a 34-channel head-stage amplifier (Gain 800, Band-Pass; 0.8 Hz–3 kHz, Triangular Biosystems, NC) inserted into the micro connector on the rat’s head. The multi-channel evoked and spontaneous signals were sampled at 16 kHz and collected in 20 s episodes into a desktop computer. In data analysis, raw signals were conditioned with an additional high-pass filter at 5 Hz and a low-pass at 400 Hz. Multiple trials were averaged to a 1 Hz stimulus in the 20 s window in order to reduce background activity against the evoked signals.

Sensory evoked potentials were elicited by a mechanical stimulation device, a tapered 1 mm cotton-tipped wood stick attached to the center of an audio speaker activated with a short pulse through a desktop computer. The mechanical stimuli were applied at a rate of one pulse per second throughout the 20 s recording episodes, bilaterally on the periphery, e.g., the left and right dorsal forearm, whiskers, face, and perioral areas. However, only ipsilateral (to injury and to the electrode implant side) dorsal hand EPs were included in this paper. All data analysis was performed in Matlab.

### Immunohistochemistry

Age matched naïve controls (*n* = 2) and 7 day post-FPI rats (*n* = 3) were anesthetized and perfused with 4% paraformaldehyde to harvest whole brains. The location of the FPI on the surface of cerebellum was marked with respect to the MEA implantation site, which was easily discernable after perfusion. The cerebellum was cut in half and then sliced in the lateral to medial direction in 50 µm parasagittal sections. The slices that contained the injury and electrode implantation regions were used for staining. Sliced sections were washed with 0.1 M phosphate buffered saline (PBS), and blocked using 10% normal goat serum in 0.3% triton in 0.1 M PBS. Then, sections were incubated overnight at room temperature with anti-CalbindinD28k antibody (MAB300, 1:1000, mouse monoclonal; Millipore) in 0.3% triton and 3% normal goat serum in PBS. Sections were reacted overnight at 4°C with Alexa 594-conjugated goat anti-mouse secondary antibody (1:500, Invitrogen) to reveal staining. Sections processed for CalbindinD28k staining were mounted on gelatin-coated slides to perform FluoroJade C staining for degenerating neurons (Neuberger et al., 2014). Sections were stained with 0.0001% FluoroJade C solution for 1 h at 4°C and cover slipped with DPX mounting media. Negative controls were routinely included in which primary antibody for CalbindinD28k was omitted. Representative images were obtained using Nikon A1R laser confocal microscope using 20× objective with identical camera settings.

## Results

### Evoked potentials

**Figure 2:**
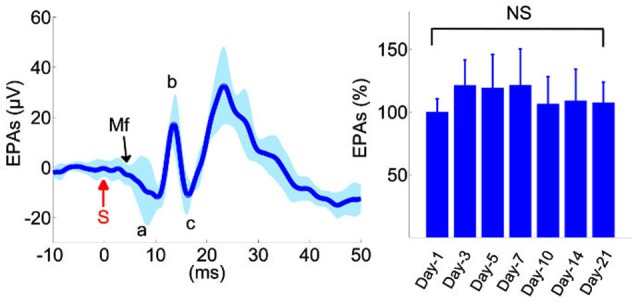
**Analysis of evoked potentials in the control animals (*n* = 6)**. Left: Evoked potential (EP) waveforms show sustained amplitudes over a 3-week period. Waveforms from 10 different recording days were superimposed from all animals. Evoked potential amplitudes (EPAs) of MF-mediated field potentials were calculated by averaging the triphasic potential amplitudes using the equation 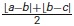. S: mechanical stimulus. Right: average MF response amplitudes from all six animals, normalized within each animal to the mean of the first day recording. None of the days were statistically different than the others (*F*_(6,59)_ = 1.41, rmANOVA, repeated measures of 6 rats,1–2 trials from each, 8–11 trials per day). Total number of trials is shown in parenthesis for each day. NS: not significant.

Evoked potentials showed reproducible waveforms in chronically implanted control animals (left plot, Figure 2), with peak-to-peak amplitudes (V_p-p_) as high as 100 µV in individual channels, while the background level of neural activity was V_rms_ = 2 µV. A typical evoked response pattern demonstrated two or three distinct field potentials. The initial negative response arrived at ~5 ms and usually measured in small amplitudes (~5–10 µV_p-p_). Conversely, subsequent evoked responses were larger positive deflections with ≥10 ms onset latencies. The first positive EP had a mean amplitude of ≥30 µV_p-p_ in the control animals (a-b, Figure 2) and it was the most reproducible component across trials and animals. This EP was presumed to originate from the local MFs in the granular cell layer and it was followed by parallel fiber synaptic activations (c, Figure 2). The largest evoked field potential was usually detected with 13–17 ms onset latency, had a mean amplitude of ≥50 µV_p-p,_ and preceded a long-lasting (50–70 ms) refractory period. This potential was not included in the amplitude analysis because of high variability between the trials in the control animals.

In order to determine reproducibility of evoked amplitudes in the control animals, EPs were collected for MF-mediated potentials (a-c, Figure 2) during the 3-week survival period and analyzed in all animals at various time points (right panel, Figure 2, *n* = 6 rats, 8–11 trials per day). None of the days was statistically different in EP amplitudes in the recording period (Repeated measures of ANOVA, *F*_(6,59)_ = 1.41), though the largest variations were observed during the first few days, e.g., ~21% on day-3 (Mann-Whitney; *n* = 9 trials, *p* = 0.06).

**Figure 3:**
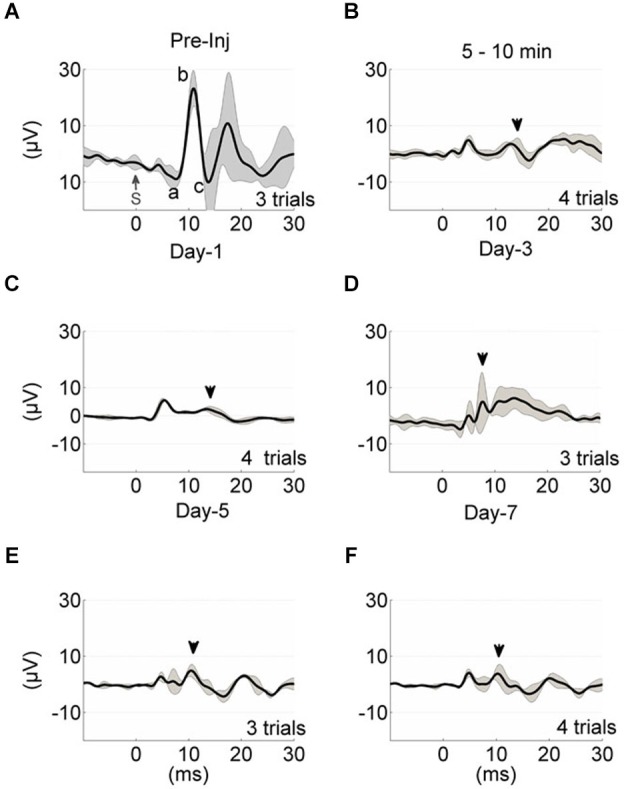
**Evoked potential waveform progression in a sample animal from the time of injury (A–F)**. Each trace represents the average of multiple trials (gray traces), and each trial is the stimulus (S, *t* = 0) trigger-average of 20 EPs. The black shade is the ± std. Mossy fiber-mediated potentials analyzed in Figure 2 are marked in similar manner (**A**, a-c; **B–F**, arrowheads). The pre-injury recordings were obtained 5–10 min before the application of FPI **(A)**. Time of recording after anesthesia was ~5 min **(A,C–F)** and 5–10 min after FPI induction **(B)**. All injury-day recordings showed great depression in the EP waveform and amplitudes. Day-3 EPs demonstrated larger amplitude variations; 5 ± 5 µV **(D)**.

**Figure 4:**
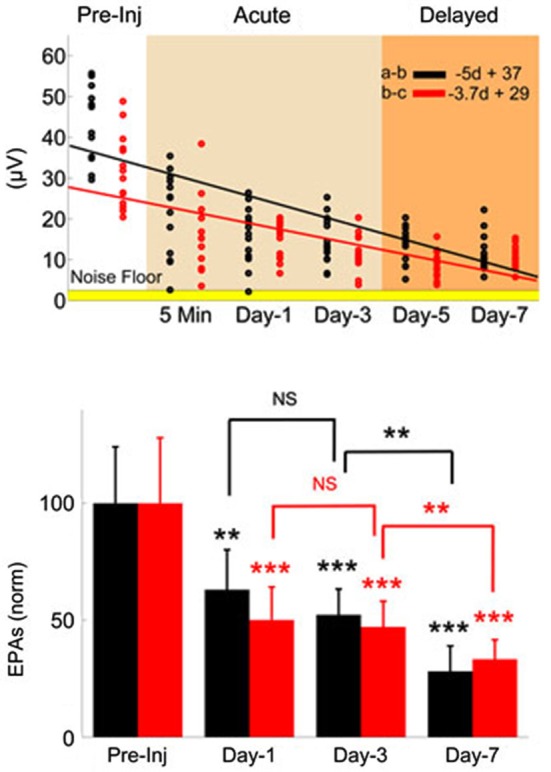
**Monitoring injury progression in EPAs of MF-mediated responses is presented on group data**. Late MF-mediated potentials, a-b and b-c (shown in Figure 3) were analyzed individually. Top: All animals as a group demonstrated nearly a linear decreasing trend by the number of days (d) after the injury (*R*^2^ = 0.47 and 0.43, respectively). Each dot in the scatter plot shows the mean of EPAs for subsequent a-b (black) and b-c potentials (red) in a different trial (*n* = 9 rats, 2–3 trials per animal on a given day). Bottom: Data shown are the mean ± std of normalized EPAs for the pre-injury and three selected days of the post-injury period. Same color representations in the scatter analysis are used here (EPA_(a-b)_; black, EPA_(b-c)_; red). All data were normalized to mean amplitude of the pre-injury recordings (*n* = 9 rats, 20 total trials). Amplitude loss reached as large as 75% at the end of the 7-day survival period. Other significances shown were calculated using repeated measures of ANOVA followed by Bonferroni correction. *P < 0.05, **P < 0.005, ***P < 0.001.

As a comparison, evoked potential waveforms are shown in an injured rat in 1-week period (Figures 3A–F). The amplitude of the MF-mediated responses declined drastically to 5 ± 2 µV (arrowhead, Figure 3B) within 10 min of injury induction from the baseline of 40 ± 5 µV (a-c, Figure 3A). There was no additional anesthesia injection at this period. Amplitude of MF-mediated EPs were diminished by ~8 fold to a value barely above the background neural activity (2 µV_rms_), while the arrival latency was preserved (10–11 ms) in these early acute responses. On the following day after injury (~20 h), similar waveform characteristics were observed in the EP signals (Figure 3C). Multiple trials indicated detectable evoked potential amplitudes (EPAs) but reduced by 8–10 times from the baseline level. Three days post-injury, EPs were regressed to about ±10 µV range (gridlines, Figure 3D). Day-3 was noted as a recession point in the EPA trend, which was consistent across all injured animals (see Figure 4). At the end of the survival period, the EPA reductions were as large as 90% of the baseline, fluctuating around ±3–5 µV (Figures 3E,F).

The MF-mediated EPAs from all injured animals (*n* = 9) are analyzed for two characteristic amplitude measures (a-b and b-c, evoked potentials, Figure 3) in Figure 4. Each dot in the scatter plot is the mean amplitude calculated from 20 spike-trigger-averaged signals in 2–3 trials on the given day (top panel). There was a clear decline in the EPAs of both components as indicated by the negative slope; V_EPA(a-b)_ = −5d + 37 and V_EPA(b-c)_ = −3.7d + 29 (d; days, Figure 4). Amplitude drops in both characteristic measures were found to be significant for each day (Day 1, 3 and 7, bottom plot) of the post-injury period against the pre-injury values (14 trials per day, *n* = 9 rats, rmANOVA, *F*_(3,52)_ = 20.52; a-b and *F*_(3,52)_ = 25.96; b-c, *p* < 0.001 ). The sharpest drops (~40%; a-b and ~50%; b-c) were observed on day-1 of injury in both measures; (Paired *t*-test; a-b; *p* = 0.0021 and b-c; *p* = 0.0007). Relatively subtle amplitude changes were seen from day-1 to day-3 (NS; a-b; *p* = 0.3282, b-c; *p* = 0.72). The EPA losses were sustained at ~50% of the baseline level on average on day-3. Prolonged recordings revealed that the greatest drops in EPAs occurred at the end of the survival period; Day-7 post-injury EPAs of a-b ~28% of the pre-injury EPAs, while it was ~33% for b-c (*n* = 9 animals; paired *t*-test, *p* < 0.0001). Statistical analysis suggested 80% power with a minimum sample size >7 animals to detect 50 ± 25% (mean ± std.) changes in normalized EPA (*α* = 0.05, two-tail).

### Spatial pattern of injury

**Figure 5:**
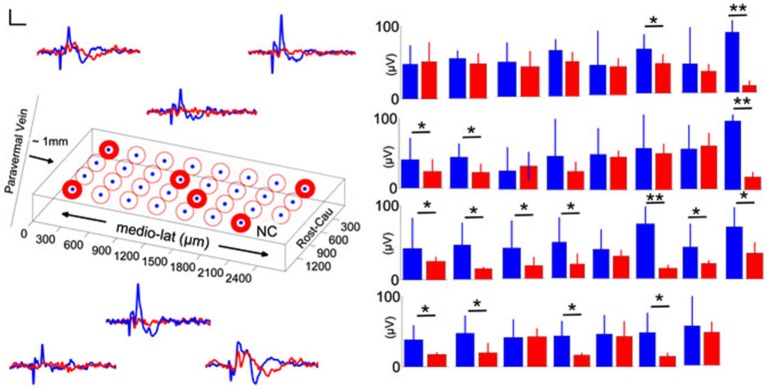
**Spatial distribution of pre-injury and day-7 (blue and red traces and bars, respectively) evoked potentials in a rat**. Left: Orientation of the MEA on the PML and averaged recordings from six representative electrode contacts. Differential changes in EPAs with respect to the contact position were noted (*n* = 2–3 trials averaged for each day in one rat). NC: no channel, scale bar = 20 µV and 20 ms. Right: Pre- and post-injury, day-7 EPA changes are illustrated for all 31-contacts. While some of the channels did not indicate a significant amplitude drop with injury, in some others the changes were drastic (9–10 trials per day; error bars show one std; paired *t*-test; *P < 0.05; **P < 0.005).

To determine whether there was a spatial variation in the altered EPs induced by injury, we analyzed the individual electrode channels by their orientation on the PML surface in one rat. Sample recordings of the pre-injury period (blue traces, Figure 5) showed different CF field potentials amplitudes (±10–20 µV) in response to the same peripheral stimulation. The spatial pattern of amplitude distribution was different after the induction of injury, i.e., each channel was affected by different amounts regardless of their initial amplitude (red traces). In some channels the amplitude decline was as large as 60–70 µV (paired *t*-test, *p* < 0.005), whereas in others as small as 5–10 µV (paired *t*-test, *p* > 0.55). Measurable changes clustered in both sagittally and transversely oriented electrode groups, however, the transverse group contained more contacts (center two rows; *n* = 11/16 channels). Interestingly, the amplitude reductions in this animal are less at the electrode sites closer to the injury point (~2 mm in rostral direction) compared to the distant contacts.

### Synchrony

**Figure 6:**
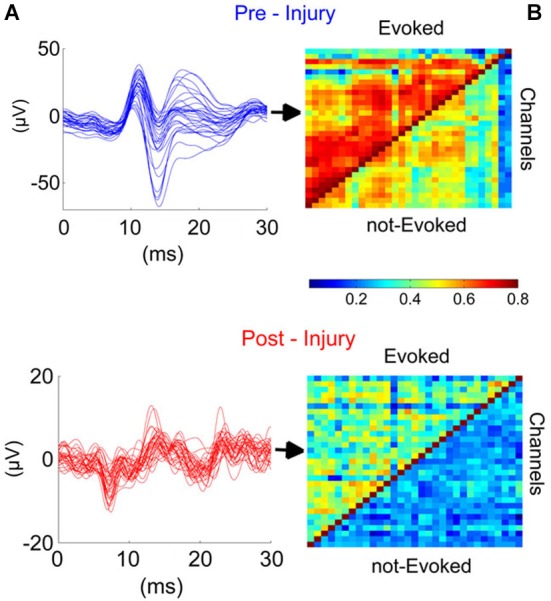
**Injury disrupted the synchrony across cerebellar cortex in the studied region (PML) during the evoked and not evoked (no stimulus) as demonstrated in this sample recording**. **(A)** Traces (Blue and red; before and after injury, respectively) illustrate the EPs for all 31-channels. High synchronization (Top) was lost after the injury (7-day FPI; bottom). **(B)** Cross-correlations between all contact pairs for pre- and post-injury signals during EPs and nEPs periods. Prior to injury, inter-channel correlations varied within *R* = 0.5–0.8 and 0.3–0.6 during EPs and nEPs recordings, respectively (Top). Cross-correlation values diminished drastically in both EPs (*R* = 0.2–0.5) and nEPs periods (*R* = 0.2–0.4) by day-7 of injury (Bottom). EPs, evoked potentials; nEPs, not-evoked potentials.

In order to evaluate the effect of injury on synchrony across the cerebellar cortex, we investigated cross-correlations between all contact pairs during evoked and spontaneous activity (not-evoked) before and after injury (Figures 6A,B). The EPs were large in amplitude and highly similar in waveform and timing across all 31-channels in the pre-injury trials (Figure 6A, top panel). Pearson correlation was as high as *r* ≥ 0.8 between some channel pairs of EP waveforms, while it varied between 0.2 ≤ *r* ≤ 0.6 during spontaneous oscillations (Figure 6B, top panel). After the injury, overall correlation for all channel pairs decreased by ~2-fold (Figure 6B, bottom panel), i.e., to ~0.2–0.3 and 0.3–0.4, respectively for spontaneous and evoked windows. Those channels that showed stronger correlations in the pre-injury trials (red squares in top panel) were also diminished to mean values of *r* = 0.3–0.4. Disruption of synchrony in evoked LFPs was clearly noticeable in the spike-trigger averaged plots of single channels (Figure 6A, bottom panel).

**Figure 7:**
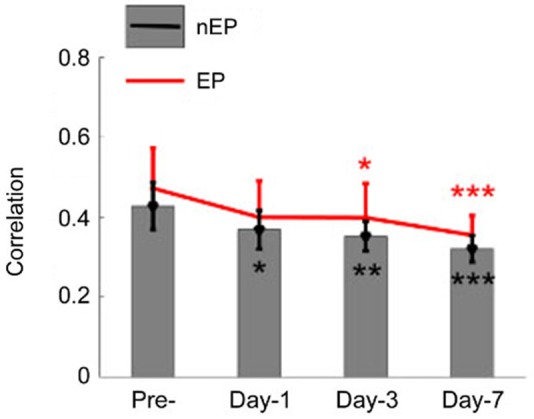
**Cross-correlations between all channel pairs were averaged in all animals and shown for the EP and not-evoked signals as in Figure 6**. A declining trend was observed in this group data after the injury (*n* = 9 rats, 20 trials per day). By the end of the survival period, the mean and std of correlations were reduced to 0.35 ± 0.04 (from pre-inj. of 0.47 ± 0.12) and 0.32 ± 0.03 (from pre-inj. of 0.43 ± 0.06) for EP and nEP, respectively. Wilcoxon-signed rank test, *P < 0.05, **P < 0.005, ***P < 0.001.

Correlation analysis was extended to all the animals at three time points after injury (*n* = 9 rats, Figure 7). Greatest reduction in mean correlation values for evoked and spontaneous recordings was noted at day-1 and day-7 of injury. At day-1 of injury, correlation value ‘*r*’ declined to 0.4 ± 0.09 from 0.47 ± 0.1 (EP-period, *n* = 9 rats; *p* = 0.055) and 0.37 ± 0.048 from 0.428 ± 0.06 (Spontaneous, *n* = 9 rats; *p* = 0.012). Correlation values exhibited greater deviations in the EP-period analysis due to existence of clustered electrode channels (see pre-injury results in Figure 6). Evoked potential correlations indicated that the first significant drops occurred at day-3 of injury, with only subtle differences from day-1 results (*p* = 0.044, *r* = 0.39 ± 0.08). Losses in averaged correlation values reached the greatest level at the end of the survival period in comparison to pre-injury values for both spontaneous and evoked period recordings. At day-7, averaged correlation was down to 0.35 ± 0.049 in the evoked-window and 0.32 ±0.033 in the spontaneous recordings (*n* = 9 rats, 20 trials per analysis; *p* < 0.001).

### Immunohistology

**Figure 8:**
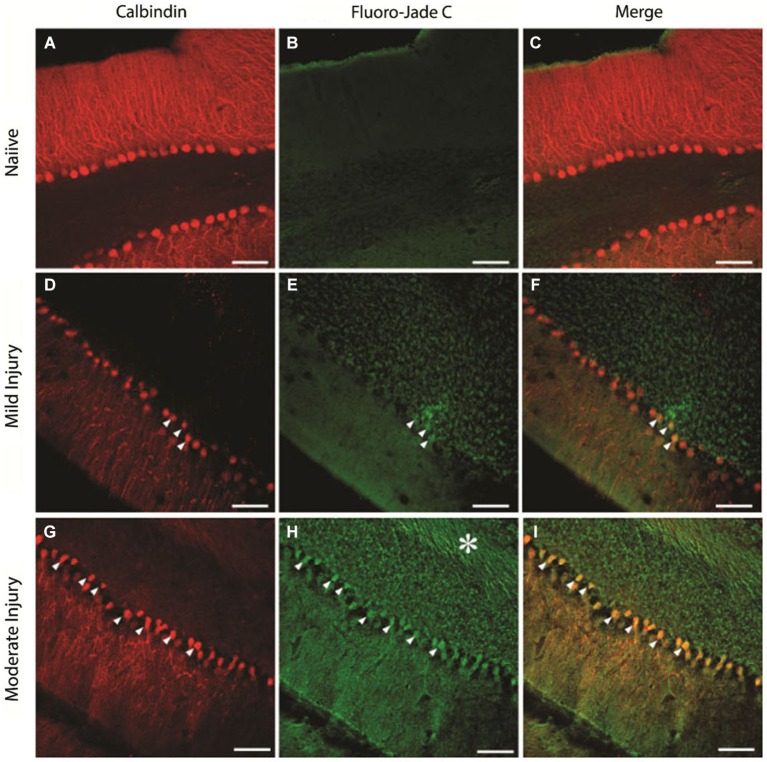
**Purkinje cell degeneration at mild and moderate levels of severity of cerebellar injury (15 psi and 25 psi)**. **(A–C)** Representative confocal images show CalbindinD28k labeled PCs **(A)** and the absence of FluoroJade C staining **(B)** in the same section from a naïve rat **(D–F)**. Images of a section from a rat 1 week after mild injury (15 psi) shows CalbindinD28k positive Purkinje cells **(D)** and the presence of a few FluoroJade C labeled cellular profiles (arrowheads in **E**). Merged image **(F)** shows that CalbindinD28k positive Purkinje cells are co-labeled with FluoroJade C (arrowheads) **(G–I)**. Representative section from a rat 1 week after moderate injury (25 psi) shows CalbindinD28k positive Purkinje cells **(G)** and extensive cellular (arrowheads) and axonal (asterisk) FluoroJade C labeling **(H)**. Merged image **(I)** shows numerous CalbindinD28k positive PCs labeled with FluoroJade C (arrowheads). Scale bar: 100 µm.

To determine the extent of cellular loss underlying the observed electrophysiological effects of injury, we performed double immunostaining of the cerebellar tissue extracted at the end of the physiological studies for expression of CalbindinD28k, a marker for PCs (Ishikawa et al., 1995) and FluoroJadeC. As expected, naïve animals showed a layer of CalbindinD28k-positive PCs (Figures 8A–C, *n* = 15 sections from three rats) with no FluoroJadeC labeling demonstrating the lack of neurodegeneration. There was a modest PC degeneration, as indicated by co-labeling of CalbindinD28k with FluoroJadeC labeled neuronal profiles in mildly injured rats (15 psi; Figures 8D–F, *n* = 6 sections from two rats). We found more CalbindinD28k expressing PCs co-labeled with FluoroJadeC in cerebellar sections from the rats injured at larger peak pressures, indicating more extensive PC degeneration (Figures 8G–I, *n* = 6 sections from one rat) than those with smaller peak pressure (Figures 8D–F). In addition to cell loss, sections from the rats subjected to injuries at higher peak pressures showed FluoroJadeC staining in the white matter tracks (asterisk in Figure 8H) suggesting the possibility of PC axonal degeneration. Remarkably, the domino-like alignment of PCs observed in naïve animals was degraded following injury at higher pressures (~25 psi). The presence of FluorojadeC positive neurons in all FPI animals indicates ongoing neuronal degeneration at 1 week.

## Discussion

### Identification of evoked field potentials

Evoked potentials have been investigated for assessment of severity in TBIs. Visual (Lachapelle et al., 2004), brainstem auditory (Soustiel et al., 1995), and sensory (Fossi et al., 2006; Amantini et al., 2009) EPs in EEG signals were proposed previously for detection of head injuries. The current study demonstrated prolonged reductions in the cerebellar EPs to hand stimulations during the 7-day period following the injury in a rat model of FPI. Characteristics (onset latencies and amplitudes) of MF- and CF-mediated cerebellar EPs were well documented by other investigators with highly reproducible results (Eccles et al., 1966a, 1967; Armstrong and Harvey, 1968; Armstrong and Drew, 1980; Atkins and Apps, 1997; Jörntell et al., 2000; Ordek et al., 2012). Our recordings in control animals (Figure 1) confirmed these reports with characteristic EPs containing mossy fiber (Brihaye et al., 1964; Eccles et al., 1967; Armstrong and Drew, 1980) and CF mediated responses (Eccles et al., 1967; Armstrong and Drew, 1980; Atkins and Apps, 1997) differentiated by their onset latencies.

Various components of surface recorded EPs were identified by the source localization in earlier reports (Eccles et al., 1967; Oscarsson, 1968; Armstrong and Drew, 1980; Atkins and Apps, 1997; Baker et al., 2001; Diwakar et al., 2011). Eccles et al. (1967) reported MF-mediated responses (MF— GCs—Parallel fibers—PCs) to have less than 5 ms onset latencies and denoted various deflections as P1, N1—N3, N4. Armstrong and Drew (1980) also identified similar MF-mediated field potentials within the same arrival latencies in response to peripheral electric stimulation in rats. In agreement to both, our control recordings indicated that the earliest noticeable evoked deflection was at ~3–5 ms following the stimulus (Figure 2), which was relatively a weak response with a negative polarity and probably a direct recording from the MFs (P1-N1). The rising edge of the subsequent evoked response was detected at 8–11 ms in our recordings. This field potential was triphasic with a positive polarity, succeeded by excitation of parallel fibers (b-c, Figure 2), and it was attributed to MF activation in the granular layer (a-b, Figure 2). Evoked potentials (a-b and b-c) had in fact the most consistent and reproducible amplitudes among all evoked responses collected in the control animals, and thus we leveraged them for monitoring the injury progression (Figures 3, 4). The onset latency of this MF field potential is slightly larger compared to the earlier reports. This may be explained by the stimulation paradigm, i.e., the location of the peripheral stimulation and the type of the stimulus. For instance, PC response latencies can increase from 6–8 ms to 7–10 ms by using tactile stimulation on the periphery instead of electrical stimulation of afferents (Bower and Woolston, 1983).

Conversely, CF-mediated potentials resembled late onset latencies (≥15 ms) in our results, which agreed with the previous reports. Pioneering studies on this subject showed that the CF activity arrives with ≥13 ms onset latencies in surface recordings (Eccles et al., 1966b; Armstrong and Drew, 1980). The delay varied between 13–19 ms in the contralateral hemisphere and between 16–22 ms in the contralateral vermis (Armstrong and Drew, 1980). Armstrong et al. (1973) also found evidence for increased PC activity with CF activation with 12–18 ms latencies compared to 4–10 ms latencies mediated by the MFs. More recently, Apps et al. demonstrated that CF response latencies to ipsilateral arm stimulation in anesthetized rats can vary between 16–26 ms when recorded from area 3 of the PML surface (Atkins and Apps, 1997). In addition to onset latencies, the CF-mediated responses can be identified by their amplitude and polarity. They are the largest positive deflections in the signals recorded from the cerebellar surface (Oscarsson, 1968; Armstrong et al., 1973) and distinguished by a refractory period up to 40 ms proceeding from the positive deflections (Armstrong and Harvey, 1968). Based on these features, we concluded that the latest EP in our recordings, which was detected at 15–20 ms in response to hand stimulation, was mediated through CF activation. The onset latency of the deflection was not the only evidence to support CF identification. The wave also resembled a strong and slow nature that succeeded a long-lasting (>50 ms) refractory period. Although the magnitude of this potential was the greatest in most of the trials, we did not include this deflection in amplitude analysis due to high variability across animals and trials. All evoked recordings presented in this particular work were obtained in anesthetized animals to avoid variations in the evoked responses due to changes in the cerebellar excitability across different awake states.

### Stability of EP recordings

Stability of EP amplitudes in the control animals clearly demonstrated that the tissue responses did not compromise the electrode array’s ability to measure reproducibility of signals during 3-week implant period (Figure 2). Pre-injury recordings in the FPI animals also served as an additional confirmation on the feasibility of this recording method of EPs (Figure 3A). The depth of ketamine-xylazine anesthesia may alter the EP amplitudes (Bengtsson and Jörntell, 2007; Ordek et al., 2012). Jörntell et al. (2000) showed the anesthesia effects on both MF- and CF-mediated responses at varying doses of ketamine/xylazine injections (Bengtsson and Jörntell, 2007). In order to minimize the effect of anesthesia depth, the timing and duration of the recording sessions from the injection of anesthesia were carefully controlled (see Section Methods). After testing various peripheral sites (whisker, forelimb, hindlimb) of stimulation, we concluded that the late MF-related response to (ipsilateral) dorsal hand mechanical stimulation was the most reproducible as a pattern in the recordings of the investigated PML region.

### FPI model

The FPI model implemented in the current study produces a combination of focal and diffuse damages, and it is widely used as an animal model of TBI (Thompson et al., 2005). It was documented that FPI can induce structural and functional changes in the cerebellar cortex even at remote locations (Ai et al., 2007). This allowed us to apply the injury at a different location from the site of electrode implant without disturbing the electrode-tissue interface. Immunohistochemical analysis further verified that the injury was spread to remote locations within the ipsilateral cerebellum.

### Primary vs. delayed injury phases

To investigate the relation between electrophysiological signals and the cerebellar insults, we demonstrated EPA changes as early as 5 min post-injury to 1 week in anesthetized rats, following FPI. Immediate recordings after trauma indicated substantial depression of the EP pattern as a whole; an effect that must be directly linked to initial impact of injury, e.g., tissue and/or blood vessel damage, and intracranial pressure elevation (Gaetz, 2004; Cernak, 2005). Interestingly, early arriving EP (<5 ms), presumably the direct MF activation, starts to increase in magnitude during the post-injury phase, which may be explained by hyper excitability of MFs as reported earlier (Ai and Baker, 2004). Monitoring the progression of injury-related changes at such detail can provide further insights about the course of the injury progression, which has been reported to present two main phases; primary and delayed-mechanisms (Doppenberg et al., 2004; Andriessen et al., 2010).

Progressive PC losses were documented in short (hours) as well as longitudinal (days—weeks) studies of immunohistology (Fukuda et al., 1996; Mautes et al., 1996). In support of this finding, MF and CF mediated EPAs of this study monotonously decreased in the post-injury period (Figure 3). At a closer evaluation, we quantified the EPA alteration in the late MF-mediated responses by analyzing in two separate EPs; a-b and b-c. Alterations in both response amplitudes were found to be very similar but not identical.

We determined that the largest drops were observed in the acute period for both amplitude measures between immediately after the injury to day-1. The EPA drops were more significant in the b-c segment (~50%) compared to the a-b potential (~37%) in this period. The second most drastic drop was noted from day-3 to day-7 (24% and 14%, respectively), which was termed as the delayed injury period in other reports (Sato et al., 2001). The two phases with distinct characteristics suggest two different injury mechanisms involved. Baker and colleagues concluded that day-3 is a critical time point in the course of injury (Ai and Baker, 2004; Ai et al., 2007). They found majority of cell deaths within the next 24 h after FPI, and the second wave of injury effect was delayed until day-3 (Ai et al., 2007).

### Spatial differentiation

Fluid percussion injury induces a combination of focal and diffuse type of injuries (Potts et al., 2009). We found spatially varying degrees of EPA reduction across the PML surface covered by the electrode array (Figure 5). Somatotopy of the PML in the rat cerebellum was investigated in several reports including ours (Bower and Woolston, 1983; Atkins and Apps, 1997; Ordek et al., 2012), though there is no consensus regarding a single somatotopy in the cerebellum. In the animal shown in Figure 5 (blue traces) the EP responses collected from the lateral side of the PML were relatively larger. Following FPI induction, EPAs were affected differentially across the PML surface without a certain directional preference. Interestingly, the smallest amplitude changes were observed on the most rostral contacts of the MEA closest to the injury site. This spatial differentiation supports our premise that the evoked amplitude changes are not due to some mechanical perturbation of the MEA by the fluid pressure wave at the time of impact, but because of damage to the underlying neural structures.

### Synchrony

Local field potentials emerge from the synchrony of a large population of neural components underneath the electrode contacts. Synchrony is observed at multiple levels of cerebellar cortex at various frequency bands. Low-frequency band (1–4 Hz) oscillations in the molecular layer were proposed to originate from the inferior olive and modulate the PC activity via the CF afferents (Lang et al., 2006). Whereas, theta and beta band oscillations are generated in the granular layer by MF activations (Hartmann and Bower, 1998; D’Angelo et al., 2001). In contrast to the deeper layers, the neurons in the molecular layer of the cerebellar cortex are capable of oscillating at higher frequencies, for instance at 30–80 Hz due to the interneuronal feedback mechanism in the molecular layer (Middleton et al., 2008) and 160–260 Hz in the Purkinje layer via axon collaterals (de Solages et al., 2008). Presumably, these oscillations originate from different neural structures, which have specific spatial alignments in the cerebellar cortex. For instance, it is possible to record sagittal synchrony in lower frequencies (1–4 Hz) that is mediated by the CFs (Lang et al., 2006) while the interneurons in the ML exhibit higher frequency oscillations (30–80 Hz) in the transverse plane (Middleton et al., 2008).Considering that the surface recordings with ball electrodes are able to detect even the deep MF related potentials, the subdural MEAs should be able to detect synchronous activities from various layers of the cerebellar cortex.

Although the synchrony in cerebellar cortex has been investigated for decades, there are just a few reports on the spatial aspect of these events. De Zeeuw et al. (2011) reviewed the spatiotemporal aspects of cerebellar oscillations in a recent report. They argued that complex spike synchrony can be observed between PCs that are separated up to ~500 µm in the parasagittal zones and which is consistent throughout the cerebellar cortex. In contrast, simple spike synchrony doesn’t indicate any particular directionality. In the flocculus, simple spike synchrony is in the same orientation of complex spike spatial pattern (parasagittal), whereas the synchrony is mainly oriented along the transverse plane in Crus 2 and in the PML. de Solages et al. (2008) showed the high-frequency oscillations (~200 Hz) by using tetrode and multi-site recordings in both anesthetized and awake animals. They also showed that the synchrony at this frequency band extended as far as 375 µm across the electrodes aligned in the transversal orientation. Furthermore, their report suggested that there was a correlation between the oscillations obtained from different layers of cerebellar cortex, where molecular and Purkinje layer recordings displayed peak coherences. Similarly, Courtemanche et al. (2013) documented the ~200 Hz oscillations in the Purkinje cell layer of anesthetized rats with metal electrodes separated by 500 µm.

In addition to *in vivo* studies, pathological findings also indicated the large scale synchrony in the cerebellar cortex. In the experiments on cerebellar mutant mice, high-frequency synchrony (>150 Hz) was shown in the zones up to 1 mm (Servais and Cheron, 2005). Some of these oscillations were also noted in calretinin/calbindin mutant mice along the parallel fiber orientations ranging up to 2 mm (Servais et al., 2004; Cheron et al., 2008). These reports support the spatial synchrony that we observe in our recordings, although the area covered by our MEA was unprecedented in size (300 µm–2100 µm).

The effect of anesthesia on spontaneous recordings as well as EPs is a concern raised by a number of investigators in the past. Cheron and his colleague (Servais and Cheron, 2005) compared the differential effects of two different anesthesia regimens (ketamine and pentobarbitone) on LFPs. They found that ketamine, an NMDA antagonist, depresses the LFP oscillations with PC desynchronization, while pentobarbitone, which targets the GABA_a_ receptors, caused slight changes in PC synchrony. In the cerebellum, excitatory networks such as the MF-GC-PFs pathway use the NMDA receptors, whereas inhibitory signaling is mediated by GABA_a_ receptors through the PCs and molecular layer interneurons. Therefore, using different anesthesia regimens could have different effects on the neural activity by selectively targeting different synaptic mechanisms. Another critical factor in anesthesia is the time delay allowed before data collection. Jörntell and his colleague (Bengtsson and Jörntell, 2007) reported that ketamine-xylazine (20:1) depressed both MF and CF responses significantly for 10 min after the injection. Similarly, LFP oscillations in the cerebellum exhibited sustained depressions for 5–10 min after anesthetic injection (Servais and Cheron, 2005). Although the recovery time was dose dependent, we observed that the delay allowed between the injection and recordings can be used to control the anesthesia level in a reproducible manner, and thus obtain stable recordings.

Our findings suggested that there was a significant correlation loss in all electrode groups starting day-1 of injury. The correlation test applied to the EEG signals after head injuries is a diagnostic technique that has been used over decades. Thatcher et al. (1989) reported coherence changes across short-distance in different frequency bands after mild injuries. Our findings indicated progressive reductions in correlation values during spontaneous as well stimulated periods (Figure 7). Interestingly, correlation loss paralleled the decline in the EPAs. Both measures exhibited similar trends, i.e., greater losses at day-1 and then from day-5 to day- 7, but only subtle changes from day-1 to day-3. This suggests that the injury did not only affect the number of PCs that are firing in synch through MF or CF activations, hence the EPA loss, but also the connectivity between spatially distant zones (within 2 mm^2^) was disrupted.

### Immunohistology

We included immunohistological analysis into this study primarily for two reasons; first, to verify that an injury-related neuronal degeneration was produced by direct-FPI to the cerebellum. We used double immunostaining; CalbindinD28k and Fluoro-Jade C to determine the neuronal subtype that was injured. Fluoro-Jade markings showed irreversible cell deaths as delayed as 1 month of injury induction (molecular layer neurons and PCs) in the cerebellum by earlier reports (Sato et al., 2001). Hallam et al. (2004) also showed Fluoro-Jade C positive degenerating neurons at different time points (24 h, 48 h and 7-days) of the FPI in the rat cerebellum, which was correlated with motor behavioral deficits. Second, we aimed to evaluate the sensitivity of the electrophysiological parameters to detect neural damage due to FPI compared to immunostaining. Our results indicated that the subdural MEA recordings were able to glean valuable information about the injury at peak pressures as mild as 15 psi, an injury pressure that resulted in minimal neuronal degeneration by immunohistology (Figures 8D–F) and no overt behavioral deficits.

## Conclusions

Here we presented data showing the feasibility of monitoring injury related changes in the cerebellar cortex using EPs recorded with subdurally implanted MEAs. Changes in peripherally evoked signal amplitudes were detected by 5-min post-injury recordings, and monitored periodically in the following 7 days. Our results also presented evidences showing that the decline of inter-contact correlations followed a similar trend to the evoked amplitudes in the 1 week post-injury period. Immunohistological results confirmed the cellular degenerations in the targeted cerebellar area as a result of injury. Overall, electrophysiological monitoring using MEAs is a promising technique to study the progression of neuronal degeneration in animal models of injury without the need of terminating experimental subjects at various time points in the study.

## Conflict of interest statement

The authors declare that the research was conducted in the absence of any commercial or financial relationships that could be construed as a potential conflict of interest.
